# Constitutive homologous expression of phosphoglucomutase and transaldolase increases the metabolic flux of *Fusarium oxysporum*

**DOI:** 10.1186/1475-2859-13-43

**Published:** 2014-03-20

**Authors:** George E Anasontzis, Elisavet Kourtoglou, Diomi Mamma, Silas G Villas-Boâs, Dimitris G Hatzinikolaou, Paul Christakopoulos

**Affiliations:** 1 Microbial Biotechnology Unit, Sector of Botany, Department of Biology, National and Kapodistrian University of Athens, Athens, Zografou, Greece; 2 BIOtechMASS Unit, Biotechnology Laboratory, School of Chemical Engineering, National Technical University of Athens, Athens, Zografou, Greece; 3 Centre for Microbial Innovation, School of Biological Sciences, The University of Auckland, Auckland, New Zealand; 4 Biochemical and Chemical Process Engineering, Division of Sustainable Process Engineering, Department of Civil, Environmental and Natural Resources Engineering, Luleå University of Technology, Luleå SE-97187, Sweden; 5 Current address: Industrial Biotechnology, Department of Chemical and Biological Engineering, Chalmers University of Technology, Gothenburg, Sweden

**Keywords:** Phosphoglucomutase, Transaldolase, Metabolic flux, Fungi, PPP, Glycolysis, Metabolic engineering

## Abstract

**Background:**

*Fusarium oxysporum* is among the few filamentous fungi that have been reported of being able to directly ferment biomass to ethanol in a consolidated bioprocess. Understanding its metabolic pathways and their limitations can provide some insights on the genetic modifications required to enhance its growth and subsequent fermentation capability. In this study, we investigated the hypothesis reported previously that phosphoglucomutase and transaldolase are metabolic bottlenecks in the glycolysis and pentose phosphate pathway of the *F. oxysporum* metabolism.

**Results:**

Both enzymes were homologously overexpressed in *F. oxysporum* F3 using the *gpd*A promoter of *Aspergillus nidulans* for constitutive expression. Transformants were screened for their phosphoglucomutase and transaldolase genes expression levels with northern blot. The selected transformant exhibited high mRNA levels for both genes, as well as higher specific activities of the corresponding enzymes, compared to the wild type. It also displayed more than 20 and 15% higher specific growth rate upon aerobic growth on glucose and xylose, respectively, as carbon sources and 30% higher biomass to xylose yield. The determination of the relative intracellular amino and non-amino organic acid concentrations at the end of growth on glucose revealed higher abundance of most determined metabolites between 1.5- and 3-times in the recombinant strain compared to the wild type. Lower abundance of the determined metabolites of the Krebs cycle and an 68-fold more glutamate were observed at the end of the cultivation, when xylose was used as carbon source.

**Conclusions:**

Homologous overexpression of phosphoglucomutase and transaldolase in *F. oxysporum* was shown to enhance the growth characteristics of the strain in both xylose and glucose in aerobic conditions. The intracellular metabolites profile indicated how the changes in the metabolome could have resulted in the observed growth characteristics.

## Background

The first generation biofuels, produced usually from the edible parts of crops, such as wheat and corn, are not considered as a sustainable solution to the energy problem. Not only they fail to solve oil dependency, but actually require more energy for their production than the amount intended to produce [1]. The bioconversion of plant biomass to first generation biofuels has already been in use widely in Brazil, where the high synthesis of sucrose from the abundant tropical sugar cane is utilized [2], and the U.S., where the starch obtained from corn is hydrolyzed before the yeast fermentation takes place [3]. Second generation biofuels, on the other hand, are produced by the conversion of lignocellulose and utilize the whole biomass of plants.

Lignocellulosic compounds derive from both the woody and non woody plants and consist of a major source of organic renewable material. They contain a mixture of carbohydrate polymers (cellulose and hemicelluloses) and lignin and due to their properties, they are substrates of significant biotechnological value [4]. Lignocellulose is abundant in agricultural and forestry residues as well as in the waste streams of pulp and paper and other industries and could be used as a sustainable and low cost source of fermentable sugars for supplying the ethanol production process [5].

*Saccharomyces cerevisiae* is the major microorganism used for ethanol production, due to its high metabolic rate and ethanol productivity. Despite the efforts to increase its fermentation capability for xylose [6] and several successful efforts to express cellulases [7,8] and xylanases [9,10] genes in *S. cerevisiae*, no such strains have been applied in a commercial scale. At this level, saccharification is usually performed enzymatically in a step preceding the fermentation. But evidently, there is high interest in the development of microorganisms that possess the ability to grow and break down the substrate and form products with useful properties in a consolidated bioprocess from crude plant biomass [11].

*Fusarium oxysporum* is among the few species that have been reported to bear the ability to ferment cellulose and hemicelluloses to ethanol, in a one-step process [12,13]. *F. oxysporum* produces a wide range of cellulases and xylanases [14-16] and can ferment not only D-xylose, but cellulose and hemi-celluloses as well, and therefore, the separate enzymatic hydrolysis of the crude biomass can be omitted. Also, it is relatively tolerant on sugars and ethanol [13]. Its main disadvantage, compared with yeasts, is the low conversion rate [13].

**Figure 1:**
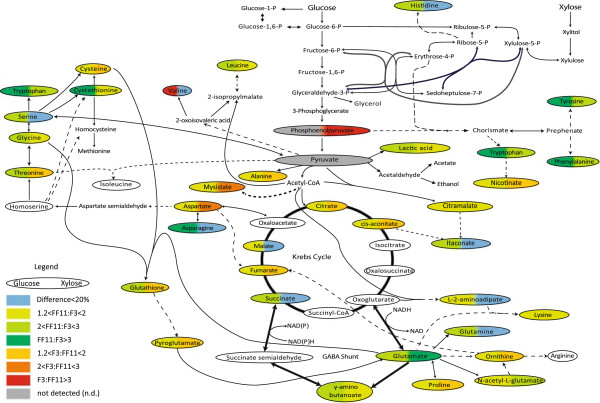
**Schematic demonstration of the relative intracellular concentrations of amino and non-amino organic acids during aerobic growth of *F. oxysporum *F3 and FF11 strains on glucose and xylose.** Single lines: One enzyme reaction; dashed lines: several reactions; Colours on the left and right correspond to the difference with glucose and xylose, respectively, as substrate. Red: F3:FF11 > 3; Orange: 2 < F3:FF11 < 3; yellow: 1.2 < F3:FF11 < 2: Dark green: FF11:F3 > 3; Green: 2 < FF11:F3 < 3; Light green: 1.2 < FF11:F3 < 2: blue: difference smaller than 20%; grey: not detected.

The strain F3 of *F. oxysporum* specifically is a high cellulase-secreting microorganism, whose metabolic flux has been extensively studied [12,17-20]. In previous studies, *F. oxysporum* F3 had accumulated high levels of glucose-1,6-diphosphate (G-1,6-2P), at the last sample taken just before glucose exhaustion in both aerobic and anaerobic phases, when grown with glucose as carbon source. The fact that glucose concentration seemed to correlate to the G-1,6-2P levels had been attributed to the potentially reduced activities of phosphoglucomutase, thus indicating a bottleneck in the channeling of glucose towards cell wall biosynthesis [18], glycolysis pathway and ethanol production (Figure 1). This enzyme is converting glucose-1-phosphate (G1P) to glucose-6-Phosphate (G6P) and vice versa, with a ping pong mechanism through G-1,6-2P [21]. Also, sedoheptulose-7-phosphate (S7P) had been detected at elevated levels, when grown in glucose-xylose mixtures, during glucose consumption phase and xylose consumption phase under oxygen-limiting conditions [17]. Similar effect has not been observed in *Saccharomyces cerevisiae*[22]*.* Also, in *F. oxysporum* F3, erythrose-4-phosphate (E4P) had accumulated during xylose consumption [17]. Overexpression of the native transaldolase of *S. cerevisiae* in a xylose fermenting strain, improved aerobic growth substantially [23] and enhanced anaerobic xylose metabolism [24]. Also, Fan et al. reported that the overexpression of two transaldolases, from *Pichia stipitis* and *S. cerevisiae,* in *F. oxysporum* had increased the xylose consumption and ethanol production when grown with glucose and xylose as carbon sources [25,26].

In the present investigation, the homologous transformation of *F. oxysporum* with the phosphoglucomutase and transaldolase genes, under constitutive regulation, was achieved and the transformants were examined for their phosphoglucomutase and transaldolase activities under aerobic conditions. Among the strains that exhibited higher activities of both enzymes, FF11 was subsequently studied in batch bioreactors for its growth characteristics on glucose or xylose.

## Results and discussion

### Strain selection

Eleven hygromycin resistant strains were selected from the co-transformation of *F. oxysporum* F3 with the plasmids pBlPGM-hyg and pBlTAL-hyg. The insertion of *pgm* and/or *tal* was confirmed with PCR and Southern blot analysis, using the *pgm* and *tal* gene, respectively, as probes.

**Figure 2:**
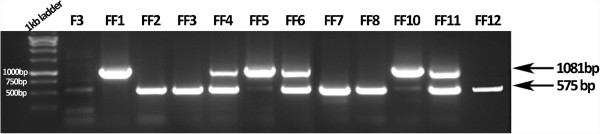
**Agarose gel electrophoresis of the PCR products, amplified with the prF, prgpmR and prtalR primers, for the selected transformant strains, as well as for the wild type (F3).** The fragments of 782 bp are specific to phosphoglucomutase, while the fragments at 1081 bp are specific for transaldolase. Strains FF1, FF5 and FF10 have incorporated the *tal* gene controlled by the promoter of *gpd*A, while strains FF2, FF3, FF7, FF8 and FF12 have incorporated the modified gene *pgm.* Strains FF4, FF6 and FF11 seem to have incorporated both plasmids.

The electrophoresis in agarose gel of the PCR products, amplified with appropriately designed primers targeting part of the construct sequence which is not present in the wild type strain, provided an indication of the construct insertion into the *F. oxysporum* genome (Figure 2).

**Figure 3:**
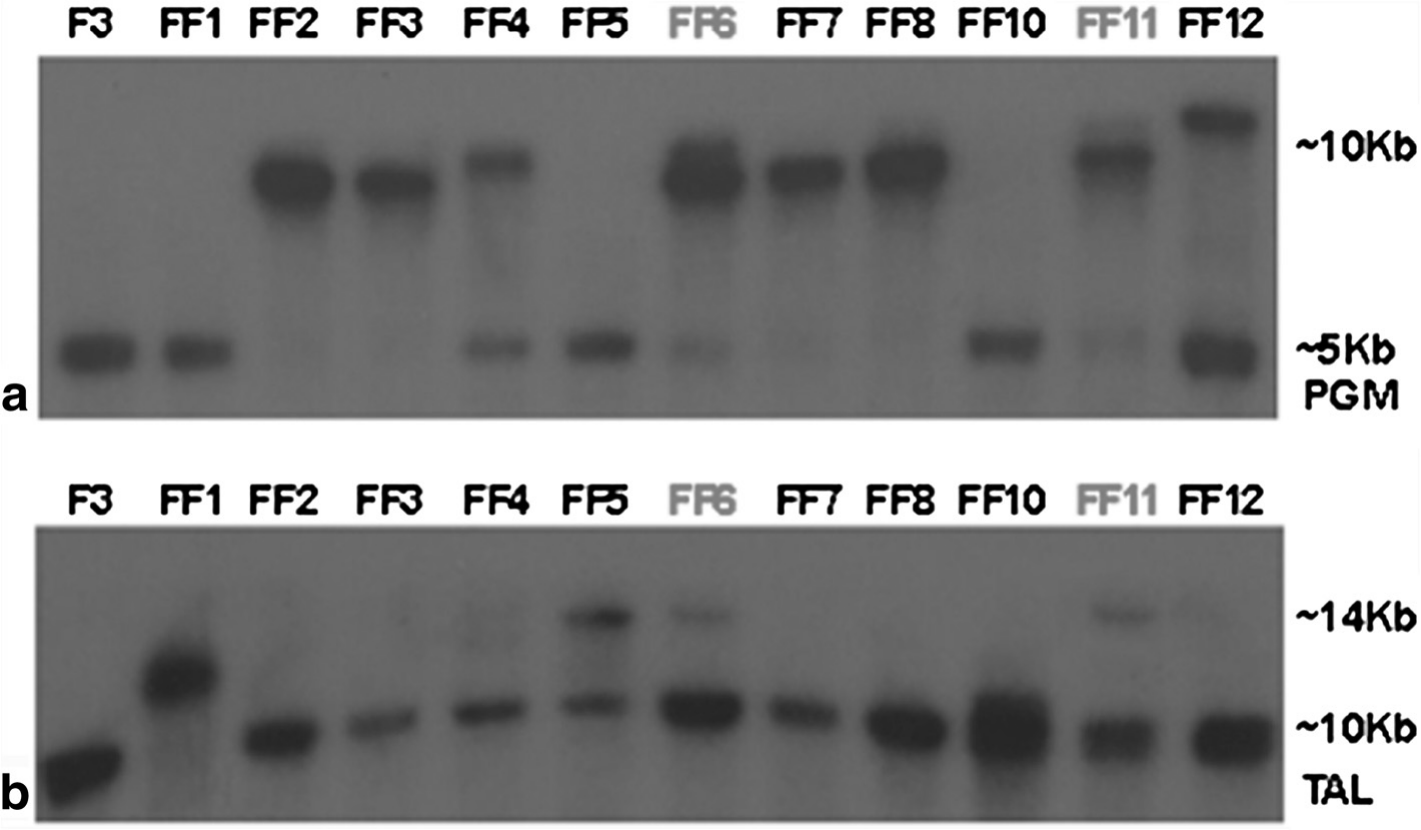
**Southern blot analysis of the eleven transformed strains, in comparison to the wild type strains (F3), using (a) *pgm *and (b) *tal *as probe.** The bands at 5 Kbp for phosphoglucomutase and 10 Kbp for transaldolase correspond to the native gene DNA fragment after fractionation of the genomic DNA with XhoI. The larger bands correspond to the whole plasmid being incorporated in the genome in the same (10 Kbp and 14 Kbp respectively) or different positions.

Southern blot analysis indicated that the original *pgm* gene was replaced in the tranformant strains FF2, FF3, FF7 and FF8 by the reconstructed one. Strains FF4, FF6, FF11 and FF12 had at least one more DNA fragment hybridized with the *pgm* DNA probe, which corresponds to the inserted construct (Figure 3). Also, only in the FF1 strain, the original transaldolase seems to have been replaced by the recombinant one. Transformants FF5, FF6 and FF11 had at least two fragments hybridized with the *tal* probe, as opposed to one for the wild type (F3). The constructs of both *pgm* and *tal* have been inserted only in the FF6 and FF11 transformant strains, in addition to the native genes.

**Figure 4:**
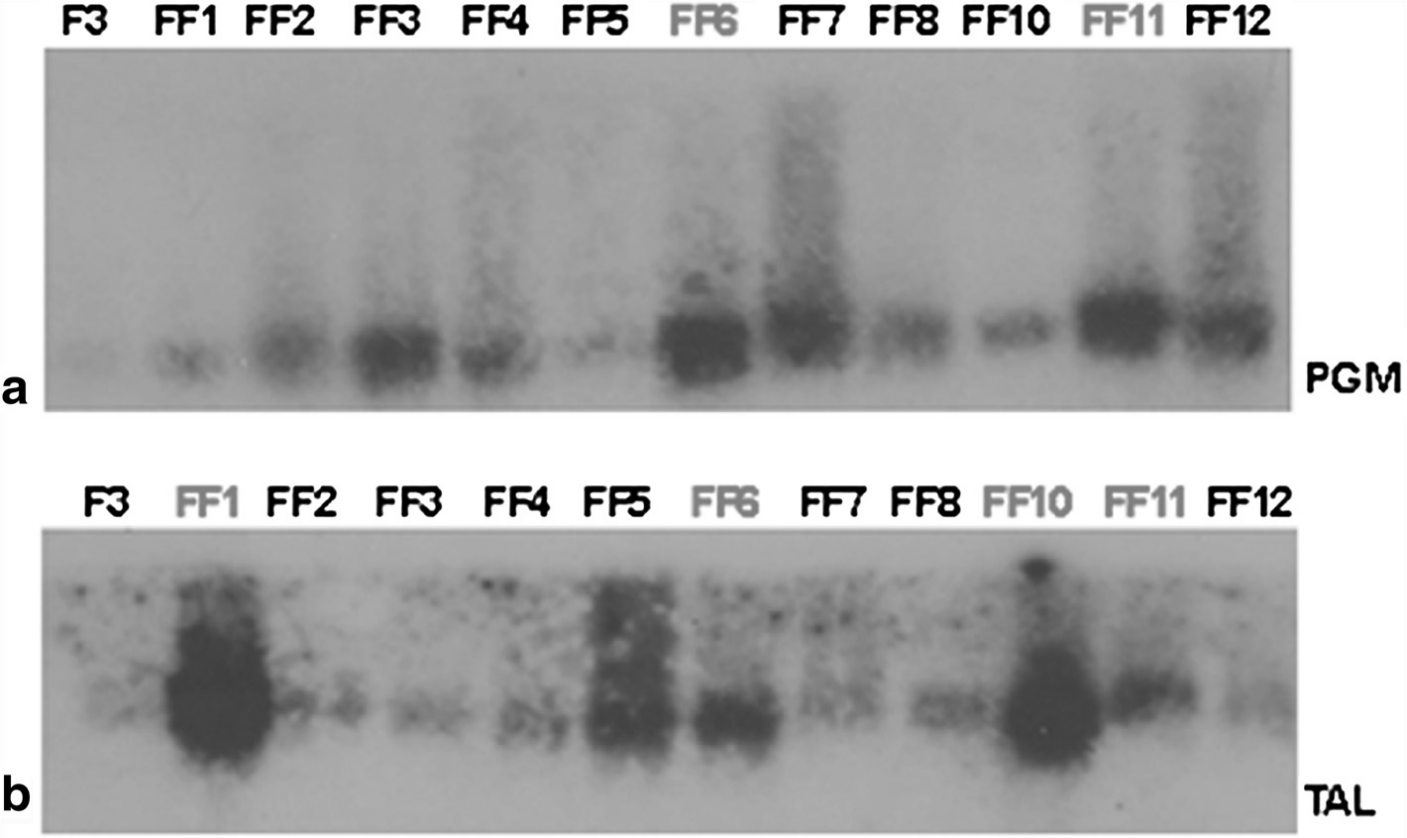
**Northern blot analysis of the eleven transformant strains, in comparison to the wild type (F3), using (a) *pgm *** **gene and (b) *tal *** **gene as probes.**

Northern blot analysis (Figure 4 and Additional file 1: Table S1) indicated which strains exhibited the highest transcription levels for the *pgm* and *tal* genes, compared to the wild type F3. It is evident that strains FF3, FF6, FF7, FF11 and FF12 had the highest transcription levels of *pgm*, whilst strains FF1, FF5, FF8 and FF10 had lower *pgm* transcription levels, compared to the wild type, confirming the Southern blot analysis results. Respectively, only the strains that had integrated the constructed *tal* gene (FF1, FF5, FF6, FF10 and FF11) according to Southern blot analysis, exhibited higher transaldolase transcription levels.

**Figure 5:**
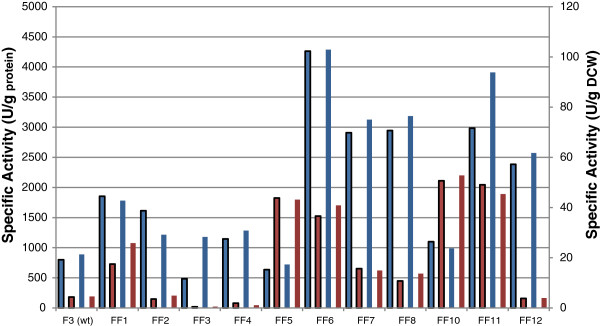
**Specific activity of phosphoglucomutase and transaldolase for the transformed strains, compared to the wild type (F3), when grown in liquid cultures with glucose as carbon source.** Strains FF6 and FF11 demonstrated high activity levels for both enzymes. All assays were performed in duplicate. Standard deviation was lower than 3%.  PGM specific activity (U^-1^ g^-1^ protein),  TAL specific activity (U^-1^ g^-1^ protein),  PGM specific activity (U^-1^ g^-1^ DCW),  TAL specific activity (U^-1^ g^-1^ DCW).

The results from the enzymatic assays are in accordance to the transcription levels. Strains FF1, FF5, FF6, FF10 and FF11 showed up to ten times higher specific activity of transaldolase compared to the wild type (F3). Phosphoglucomutase specific activity was up to almost five fold higher in the transformants FF1, FF6, FF7, FF8, FF11 and FF12. It should be mentioned that, although the extraction methodology is adequate and coherent for the comparison of the different strains, the protein yield as a percentage of the DCW is underestimated. Strain FF11 was chosen for batch culture studies to clarify the effect of the genetic modifications to the growth rates in glucose and xylose, as it showed high transcription and enzymatic activity levels for both enzymes (Figure 5 and Additional file 1: Table S2).

### Growth

**Figure 6:**
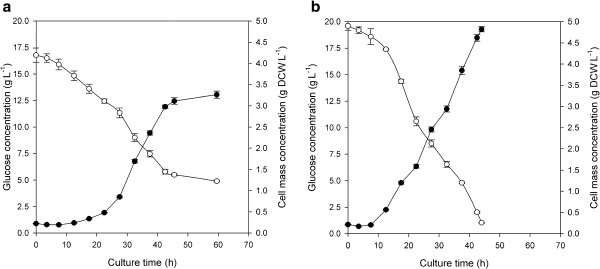
**Time course of substrate utilization and biomass formation during growth of (a) *F. oxysporum *F3 and (b) *F. oxysporum *FF11 in bioreactors using glucose as carbon source.***Symbols:* (●) Cell mass, (○) glucose.

**Figure 7:**
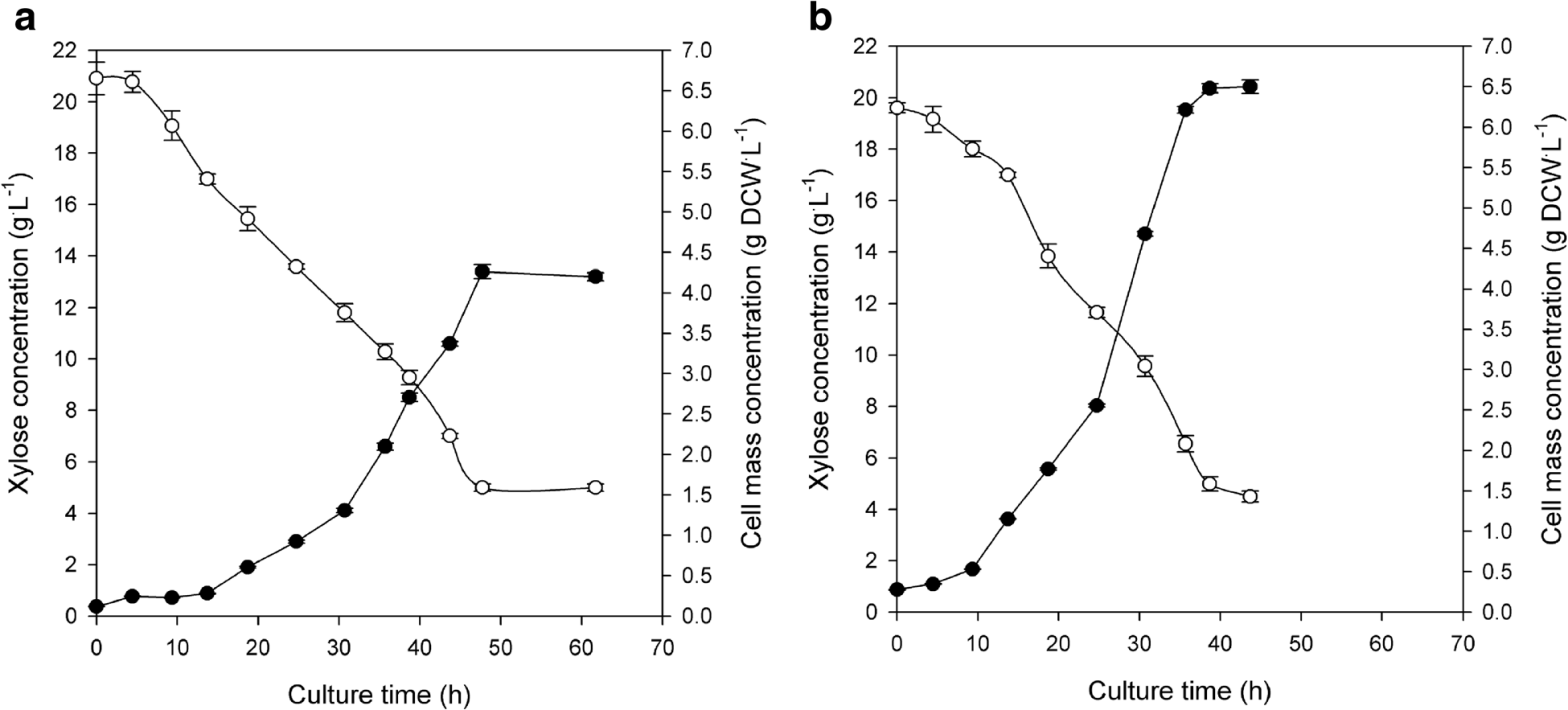
**Time course of substrate and biomass concentration during growth of (a) *F. oxysporum *F3 and (b) *F. oxysporum *FF11 in bioreactors using xylose as carbon source.***Symbols:* (●) Cell mass, (○) xylose.

Aerobic growth of both *F. oxysporum* strains (namely wild-type F3 and transformant FF11) was carried out with 1 vvm aeration. The concentrations of glucose, xylose and the (dry) cell mass during the time course of the cultivation are presented in Figures 6 and 7 respectively, while the growth characteristics of all cultures are depicted in Table 1.

**Table 1:** **Maximum specific growth rates, specific uptake rates and biomass yield coefficients during aerobic growth of strains F3 and FF11 of *Fusarium oxysporum *** **on glucose and xylose**

	***F. oxysporum *** **F3**	***F. oxysporum *** **FF11**
Glucose		
*μ*_*max*_^a^ (h^−1^)	0.096	0.119
*Y*_*x/s*_^b^ (g^.^g^−1^)	0.357	0.356
*X*_*final*_^*c*^*(g DCW*^*.*^*L*^*−1*^*)*	3.26	4.82
Xylose		
*μ*_*max*_^a^ (h^−1^)	0.086	0.099
*Y*_*x/s*_^b^ (g^.^g^−1^)	0.362	0.471
*X*_*final*_^*c*^*(g DCW*^*.*^*L*^*−1*^*)*	4.26	6.48

The standard deviation in all the cases was less than 3%.

^a^Maximum specific growth rate.

^b^Biomass yield (g dry biomass per g substrate).

^c^Final biomass concentration.

During growth on glucose the recombinant strain (FF11) exhibited higher maximum specific growth rate (0.119 h^−1^) compared to the wild type strain (0.096 h^−1^), while biomass yields for both strains were similar (0.357 and 0.356 g dry biomass per g of glucose for F3 and FF11 respectively, Table 1). At the end of fermentation (after approximately 60 h) 70% of the initial glucose was consumed by the wild type strain, while FF11 consumed almost all the available glucose within 45 h. Final biomass concentrations were 3.26 and 4.82 g DCW^.^L^−1^ for F3 and FF11 respectively. These differences in biomass concentrations are justified by the difference in the percentage of carbon source consumed. Nevertheless, the lag phase of 12.9 h exhibited by the wild type strain, compared to the half in the recombinant one, and the shorter doubling time of FF11 compared to F3, can only be attributed to the genetic modification applied.

During growth on xylose the recombinant strain (FF11) exhibited much higher biomass yield compared to F3 (0.471 g DCW^.^g_xylose_^−1^ and 0.362 g DCW g_xylose_^−1^, respectively) and slightly higher maximum specific growth rate (*μ*_*max*_) (Table 1). Furthermore, final cell mass concentration was 4.26 and 6.48 g DCW^.^L^−1^ for F3 and FF11, respectively. A lag phase of approximately 1.4 h was observed for both strains. FF11 exhibited shorter doubling time compared to F3. Xylose consumption at the end of the fermentation was approximately 75% for both strains.

As far as the maximum specific growth rates, for the wild type and the recombinant strain on both substrates (glucose and xylose), are concerned, they were found to be lower than those reported for other fungal strains, namely *Aspergillus niger* (0.22 h^−1^) [27], *Aspergillus oryzae* (0.27 h^−1^) [28], *Penicillium brasilianum* (0.18 h^−1^) [29], which might indicate that there is space for further improvement, and higher than *Penicillium brevicompactum* (0.07 h^−1^) [30].

As approach, our genetic modification resulted in a 24% increase of μ_max_, compared to, for example the 18% increase achieved by Panagiotou et al., by overexpressing an endogenous NADH kinase in *Aspergillus nidulans*[31].

Biomass yields of 0.50 g DCW^.^g_substrate_^−1^ are normally found for aerobic growth of filamentous fungi [32], but lower yields are also reported for some filamentous fungi due to extensive byproduct formation [33]. Fan et al. [25] reported lower biomass yields for wild type strain *F. oxysporum* cs28 and the recombinant strain overexpressing the *S. cerevisiae sctal.* Also, the recombinant strain had even lower biomass yields than the wild type. Although the transaldolase activity was only 0.195 times higher than the wild type, this different effect, compared to our results, can be attributed to either the different experimental layout or the increased enzyme production in our work [25]. In a very similar study, the same group showed that final biomass in a *F. oxysporum* strain overexpressing a transaldolase from *P. stipitis* was lower than that of a wild type strain during growth on xylose [26]. Both these studies were performed in flasks and in oxygen limiting conditions, parameters which could contribute to the different results. In our work, the effect of the overexpressed phosphoglucomutase cannot be ignored as a possibly significant factor for the overall higher growth rates of the recombinant strain.

### Amino and non-amino organic acids profile

The profiles of the identified intracellular amino and non-amino organic acids during growth phase, expressed as peak areas normalized by the biomass concentration are shown in Additional file 1: Table S3. The variation of each metabolite levels between the two strains and under the two different growth conditions is depicted in Figure 1.

### Glucose as carbon source

The higher concentrations of most of the determined intracellular metabolites at the end of the aerobic growth, when glucose was used as carbon source, indicate that the previously reported bottleneck [18] seems to have been bypassed, as several metabolites found after the phosphoglucomutase step have higher concentrations in the transformant strain (FF11). The higher activity of transaldolase in this strain could very well justify the higher concentrations of aromatic aminoacids, such as tyrosine, phenylalanine and tryptophan (and derivative nicotinic acid – vitamin B_3_) which are synthesized from E4P, through the shikimate pathway. In the opposite direction, transaldolase could have affected the synthesis of ribose-5-phosphate and result in higher histidine accumulation.

Under the same rational, the increased activity of phosphoglucomutase should affect the biosynthesis of alanine, valine and leucine, all derivatives of pyruvate, as well as the concentrations of the Krebs cycle intermediates and their derivatives, such as asparagine, glutamine, glutamate, lysine and proline; valine however had much lower concentrations in the recombinant strain. Other compounds found in increased concentrations in the FF11 strain are citramalic acid and its product itaconic acid, a biologically active compound [34], both derivatives of Acetyl-CoA and the Tricarboxylic Acid Cycle (TCA).

#### Glycine, serine and threonine metabolism

The higher phosphoglucomutase activity can also be indirectly accounted for the accumulation of serine and its derivative aminoacids (glycine and cysteine), as this enzyme has a key role in the glycolysis pathway. Pyruvate, which is a precursor of the above aminoacids, is being synthesized through the glycolysis pathway and it is reasonable to assume that its biosynthesis has been affected by the activity of phosphoglucomutase during glycogen degradation. This comes to agreement with the previous reported hypothesis that an increase in the activity of phosphoglucomutase in *F. oxysporum* could lead to increased activity in the glycolysis pathway [18].

#### Pyruvate metabolism

We observed an increase in abundance for leucine, but diminished amounts of valine. These aminoacids are considered to regulate their synthesis through the acetolactate synthase and high extracellular yields – especially of valine – have been connected with high phytopathogenic efficiency, through the inhibition of the respective pathways of the host plants [35,36].

#### TCA cycle

Higher levels of intracellular tricarboxylic acid cycle intermediates were detected when strain FF11 was grown on glucose, compared to wild type F3. The high succinic acid levels could be attributed to the activation of the glycoxylate pathway, as it has been proposed earlier [20,37]. This pathway is considered to be contributing to the maintenance of the redox balance [38], consuming reducing power in the form of NADH by converting succinate to succinyl-CoA through acyl-CoA transferase [37]. Similar observations have been previously reported by Panagiotou et al. [20], where a glucose defined medium supplemented with acetate led to an increase in the intracellular concentrations of succinate in the wild type strain F3. This can be attributed to the activation of the succinate propionate pathway, possibly in order to equilibrate the excess of reducing power.

We also detected higher concentrations of intracellular γ-amino-n-butyric acid in the transformant strain (FF11) during growth on both xylose and glucose as carbon sources, compared to the wild type (F3). γ-amino-n-butyric acid is an intermediate metabolite, involved in the metabolic bypassing of certain steps of the tricarboxylic acid cycle [39] via the conversion of 2-ketoglutarate to L-glutamate, instead of being oxidatively decarboxylated to succinate. The γ-amino-n-butyric pathway is the result of excessive NADH, which inhibits α-ketoglutarate dehydrogenase [40].

#### Glutamate metabolism

It has been reported that when the microorganism is grown with ammonium salt as nitrogen source, ammonium is assimilated through the action of the NADPH-dependent glutamate dehydrogenase converting α-ketoglutarate and ammonia into glutamate [41,42]. Glutamate acts as a nitrogen donor in a number of transamination reactions and it is the precursor for the biosynthesis of ornithine, arginine, proline, pyroglutamate, acetylglutamate and asparagine. The increase of the relative concentrations of glutamate and its derivatives when grown on glucose indicates that a more efficient nitrogen assimilation mechanism is taking place.

It is interesting that the pyroglutamate concentration is higher in the FF11 strain, compared to the wild type, in glucose cultures, but lower in xylose cultures. Conversion of glutamate to pyroglutamate is an important post-translational modification for the activity of several bioactive neuropeptides [43]. Pyroglutamate is part of the γ-glutamyl cycle and can be synthesized from glutathione through the combined action of γ-glutamyl-transpeptidase and γ-glutamyl cyclotransferase [44]. The function of glutathione, a non-protein thiol carrying out various functions in the fungal cells, is usually part of the cells’ response to oxidative stress due to its antioxidant properties [45]. In our study, during growth on glucose, pyroglutamate, glutamate, glutamine and glutathione concentrations were significantly higher in FF11 than in the wild type strain, indicating a more active glutathione cycle and thus either a more efficient stress response system or higher sensitivity to stress conditions.

### Xylose as carbon source

During growth on xylose, FF11 exhibited similar or even lower TCA metabolites concentrations than F3, apart from higher concentrations of γ-amino-n-butyric acid and glutamate.

In contrast with glucose, when xylose was used as carbon source, most of glutamate derivatives revealed similar or lower levels compared to the wild type, despite the fact that glutamate itself exhibits 100-times higher abundance in the transformant strain. As mentioned before, glutamate accumulation indicates a more efficient nitrogen assimilation mechanism and in this case, it possibly acts as a nitrogen reserve, with its synthesis being a possible response to high NADPH accumulation.

The cellular requirement for redox equivalents is strongly dependent on the source of nitrogen and carbon [46]. Most anabolic reductive reactions require NADPH rather than NADH as co-factor, and therefore, NADPH is mainly required in assimilation of sugars to biomass [47]. Furthermore, NADPH is used in fungi to reduce pentoses like D-xylose and L-arabinose to sugar alcohols, which are then further oxidized by NAD^+^. A major source for NADPH production is the oxidative part of the pentose phosphate pathway. Increasing the carbon flow through the PPP, primarily due to the increased transaldolase activity, could have resulted in increased NADPH availability in the recombinant strain and the subsequent increased biomass production, in the case of xylose as carbon source.

In conclusion, the constitutive homologous overexpression of phosphoglucomutase and transaldolase significantly facilitates growth on glucose and xylose of *F. oxysporum*. The recombinant strain, harbouring copies of both genes under the regulation of the constitutive promoter of *gpdA*, grew faster in both carbon sources and exhibited much higher biomass yield when grown in xylose, compared to the wild type. Examination of the differences in intracellular metabolite production between the modified and wild type strain indicated that an accumulation of glutamate was observed and its synthesis could be a response to high NADPH availability resulted by increasing the flow through PPP in the recombinant strain.

## Methods

### Microorganisms and media

Wild type F3 of *Fusarium oxysporum*[12] was maintained on PDA plates (Potato Dextrose Agar, Applichem, Germany) at 4°C, after growth for 4 days at 25°C. Plasmids pBluescript and pCSN44 were obtained from Agilent (Santa Clara, CA, USA) and the Fungal Genetics Stock Center (University of Missouri, Kansas City, USA), respectively. *Escherichia coli* strains DH5α (Invitrogen, Life Technologies, NY, USA) were used for propagation of plasmids. Plasmid purification was performed with the Nucleospin Plasmid purification kit of Macherey-Nagel GmbH & Co. (Düren, Germany).

The growth medium for DNA isolation, enzymatic activities determination and protoplast preparation (MM) consisted of (g^.^L^−1^): NaNO_3_, 3.0; KCl, 0.3; MgSO_4_^.^7H_2_O, 0.3; KH_2_PO_4_, 0.5; ZnSO_4_, 0.05; H_3_BO_3_, 0.03; MnCl_2_^.^4H_2_O, 0.01; FeSO_4_^.^7H_2_O, 0.01; CoCl_2_^.^6H_2_O, 0.005; CuSO_4_^.^5H_2_O, 0.005; (NH_4_)_6_Mo_7_O_24_^.^4H_2_O, 0.003; Na_2_EDTA, 0.1; glucose, 10.0.

The sucrose minimal medium (SMM) used for the selection of the transformants consisted of (g^.^L^−1^): sucrose, 200; KH_2_PO_4_, 1; MgSO_4_^.^7H_2_O, 0.5; KCl, 0.5; NaNO_3_, 2; glucose, 20; FeSO_4_, 0.01; agar, 4 or 15.

Media for growth in bioreactors and metabolome analyses had the following composition (g^.^L^−1^): NaH_2_PO_4_^.^2H_2_O, 7; Na_2_HPO_4_^.^2H_2_O, 9.5; KH_2_PO_4_, 1; (NH_4_)_2_HPO_4_, 10; MgSO_4_^.^7H_2_O, 0.3; CaCl_2_^.^2H_2_O, 0.3 [48]. A 5 mL mycelia and spore suspension of *F. oxysporum* from a 6 day old culture, grown on a PDA slope at 30°C, was inoculated to 500 mL Erlenmeyer flasks each containing 200 mL of the above mentioned mineral medium. Glucose or xylose (20 g^.^L^−1^ of each sugar) was used as the carbon source and pH was set to 6.0. Flasks were incubated at 30°C in an orbital shaker operating at 200 rpm for about 2 days, until the carbon source was exhausted.

The preculture was used to inoculate a 2 L stirred tank fermenter (BioFlo310, Benchtop Fermentor/Bioreactor, New Brunswick Scientific GmbH, Germany) containing 1.5 L mineral medium having the above composition. The microorganism was cultured for 48 h at 30°C and pH 6.0. Aeration level was maintained at 1 vvm, with an agitation of 200 rpm. Two cultivations were performed per strain and substrate.

### Isolation of phosphoglucomutase and transaldolase genes

Genomic DNA was isolated following standard molecular techniques [49]. Conidia of *F. oxysporum* F3 were inoculated in MM and grown at 29°C and 150 rpm for 24 h. Mycelia were then harvested, frozen in liquid nitrogen and DNA was isolated. The phosphoglucomutase gene (*pgm*) was isolated as described by Kourtoglou et al. [21]. The transaldolase gene (*tal*) was isolated from the genomic DNA, with PCR, using the Pfx high fidelity polymerase (Invitrogen, Life Technologies, NY, USA) and the appropriately constructed primers for the annotated transaldolase gene sequence (FOXG_03074.2) of *F. oxysporum* f. sp. lycopersici strain 4287 (race 2, VCG 0030) from the *Fusarium* Comparative Sequencing Project, Broad Institute of Harvard and MIT (http://www.broadinstitute.org/):

5’ –AA*GCGGCCGC*ATGTCTTCCTCTCTCGAACAGC -3’

5’ –TA*ACTAGT*TTAGGCGAGCTTCTCCTTG- 3’

This procedure resulted in a PCR fragment, comprised of the *F. oxysporum tal*, between the NotI and SpeI restriction sites (in italic).

### Plasmid construction

The vector for transaldolase overexpression was constructed by substituting the *xyl2* gene from pBlXyl [50] with the isolated *tal*, between the NotI and SpeI sites. A 2.4 kb fragment carrying the hygromycin resistance gene, isolated from plasmid pCSN44 through SalI digestion, was ligated into the plasmid, digested with XhoI. The resulting plasmid pBlTAL-hyg (Genebank accession: JF756587) and pBlPGM-hyg [21] were used for the co-transformation of the wild type *F. oxysporum* strain F3. Sequencing of the plasmids verified the presence of the corresponding gene sequences (Macrogen, Seoul, South Korea).

### Protoplast preparation and transformation

Protoplast preparation and subsequent transformation was performed as previously described [21]. Conidia from a four day PDA culture were harvested and filtered through miracloth. 10^5^ conidia were used for the inoculation of 10 mL liquid MM, incubated at 26°C and 150 rpm overnight and collected with centrifugation at 3000 × g for 5 min and diluted in 10 mL of 1.2 M MgSO_4_, pH 5.8. Glucanex (Novozymes – 0.2 g) was added and the mixture was incubated for 1.5 h at 100 rpm and 30°C. Ten mL of Sorbitol 0.6 M, MOPS 10 mM, pH 6.3, were added gently and the solution was centrifuged at 1000 × g. Protoplasts were then collected from the interface, washed in a solution of Sorbitol 1 M, MOPS 10 mM, pH 6.3, and suspended, prior to transformation, to Sorbitol 1 M, MOPS 10 mM, CaCl_2_ 40 mM.

Plasmids were diluted in 10 mM Tris buffer, pH 7.5, 1 mM EDTA and 40 mM CaCl_2_. Five μg of each plasmid solution were mixed with 100 μL protoplast solution. The mixture was kept on ice for 20 min and then 160 μL PEG4000 60% (w/v in 1 M Sorbitol, 10 mM MOPS, pH 6.3) were added. Following incubation at room temperature for 15 min, it was diluted and washed in 1 M Sorbitol, 10 mM MOPS and 40 mM CaCl_2_ solution. It was then mixed with SMM with 4 g^.^L^−1^ agar and poured on Petri dishes with SMM with 15 g^.^L^−1^ agar and 50 μg^.^mL^−1^ hygromycin. Transformants were isolated after 7 days incubation at 26°C.

### PCR analysis

Appropriate primers were designed to determine through PCR the succesful incorporation of the promoter-gene-terminator constructs in the genome. Forward primer (prF: 5’-GTTGACAAGGTCGTTGCG-3’) was located in the promoter sequence and the reverse primers were located within the sequence of the *pgm* (prpgmR: 5’-CTTCTTGACACCGTAGGCG-3’) and the *tal* (prtalR: 5’ TGTTGATGCCGTGGTCG-3’). All three primers were added in the PCR mixture. All PCR reactions were performed with Kapa Taq polymerase (Kapa Biosystems, Inc., Woburn, MA, U.S.A.).

### Southern and northern blot analyses

Southern and northern blot analyses were performed according to Sambrook et al. [49]. Genomic DNA from *F. oxysporum* wild type (F3) and transformants was digested overnight by XhoI, fractionated in a 1% agarose gel, transferred to a nitrocellulose membrane and hybridized overnight at 65°C with ^32^P-labeled probes. The latter were prepared by random primer labeling of the *pgm* and *tal* coding region of *F. oxysporum* accordingly. The membrane was washed at 65°C in 2x, 2x, 1x SSC (15 mM Sodium Citrate, 150 mM NaCl), containing 0.1% SDS for 30 min and was then exposed to X-ray film at −80°C for 3 h.

For RNA isolation, conidia were inoculated in MM and incubated for 20 h at 28°C and 150 rpm. Mycelia were harvested in miracloth, frozen in liquid nitrogen and used for RNA extraction. For northern analysis, total RNA was fractionated in 1% agarose, 10 mM orthophosphate buffer and transferred to a nitrocellulose membrane. Hybridization was performed as described above. The northern analysis results were processed using ImageJ (NIH, Bethesda, ML, USA).

### Enzymatic assays

Mycelia were harvested, grounded in liquid nitrogen and disrupted with ultrasound for 1 min (in 15 sec intervals). The phosphoglucomutase activity was determined according to Bergmeyer et al. [51], by measuring the rate of G6P production, from G1P, in the presence of G-1,6-2P. The glucose-6-phosphate dehydrogenase catalyzes the reaction of G6P and NAD towards glucolactone-6-phosphate and NADH, which is quantified by its absorbance at 340 nm (ϵ_340_ = 6290 M^−1^ cm^−1^). The assay was performed at 25°C, in 50 mM imidazolium, 10 mM MgCl_2_, 2 mM EDTA, pH 7.6, buffer (with HCl), with 6 mM G1P, 5 mM NAD, 100 μM G-1,6-2P and 1 U mL^-1^ of glucose-6-phosphate dehydrogenase.

Transaldolase activity was determined by the decrease of the optical density at 340 nm, as a result of the oxidation of NADH to NAD^+^. Transaldolase catalyzes the conversion of E4P and fructose-6-phosphate to S7P and glyceraldehyde-3-phosphate. The latter is subsequently converted to dihydroxyacetone-phosphate, through the action of triose phosphate isomerase, and finally to glycerol-phosphate by glycerol-phosphate α-dehydrogenase. The assay was performed at 25°C in 50 mM imidazolium buffer, pH 7.5, with 5 mM fructose-6-phosphate, 100 μM erythrose-4-phosphate, 100 μM NADH and 5 Units each of triose phosphate isomerase and glycerol-phosphate dehydrogenase. Reactions were performed in a final volume of 250 μL, using a Hitachi U-2900 spectrophotometer equipped with an electronic electrostatted cell holder.

### Protein determination

Protein concentration was determined according to Bradford (1976), using bovine serum albumin as standard.

### Cell and sugar concentration

Cell concentration was determined by measuring the optical density of the culture with a spectrophotometer (Hitachi UV 1100, Japan) at 600 nm, then converting to dry cell weight per liter broth (g DCW L^-1^) based on predetermined calibration curve between OD and DCW concentration.

For sugar analysis, culture samples were centrifuged at 4°C and 10.000 × g to remove the mycelium. Glucose and xylose, were analyzed by column liquid chromatography (CLC). A Jasco (PU-987) HPLC system equipped with an ion moderated partition chromatography column, Aminex HPX-87H (Bio-Rad), was used together with a Waters (410) refractive index detector. The flow rate of the mobile phase consisting of 5 mM H_2_SO_4_ was set to 0.6 mL^.^min^−1^ and the temperature to 45°C. All measurements are the result of technical duplicates from both identical fermentations (biological duplicates).

### Quenching and sample analysis

Sampling, quenching, extraction, sample derivatization and analysis of amino and non-amino organic acids were performed according to Smart et al. [52]. Samples (50 mL) of the fungal cultures were taken at the last sampling point (45 h for FF11 and 60 h for F3), just before the depletion of the carbon source and after the culture had shifted to the stationary phase. Triplicate samples (50 mL) were rapidly filtered under vacuum (Millipore, U.S.A., 0.2 μm pore size and 45 mm diameter), quickly washed with cold saline solution (4°C) and quenched in cold methanol water (1:1 v/v) at −30°C as described by Smart et al. [52]. 1 mL of the filtrate was supplemented with 0.2 μmol 2,3,3,3-d_4_-alanine and stored at −80°C until analysis of extracellular metabolites. The intracellular metabolites were extracted from the quenched cell pellets using cold methanol water and freeze-thaw cycles following the protocol described by Smart et al. [52]. The Internal standard 2,3,3,3-d_4_-alanine (0.2 μmol/sample) was added to each sample before extraction. The intracellular metabolite extracts and 1 mL of spent culture medium containing extracellular metabolites were freeze-dried (BenchTop K manifold freeze dryer, VirTis) before chemical derivatisation.

The freeze-dried samples were derivatized using the methyl chloroformate (MCF) method according to the protocol described by Smart et al. [52]. In summary, the freeze-dried samples were resuspended in 200 μL of sodium hydroxide solution (1 M) and transferred to a silanized glass tube, then mixed with 167 μL of methanol and 34 μL of pyridine. The derivatization began by adding 20 μL of MCF followed by vigorously mixing for 30 sec, and then a further 20 μL of MCF was added followed by vigorously mixing for 30 sec. To separate MCF derivatives from the reactive mixture, 400 μL of chloroform was added and vigorously mixed for 10 sec followed by the addition of 400 μL of sodium bicarbonate solution (50 mM), and mixing for an additional 10 sec. The aqueous layer was removed and dehydrated with anhydrous sodium sulphate before samples were transferred to GC-MS vials.

The MCF derivatives were analysed in an Agilent GC7890 system coupled to a MSD5975 mass selective detector (EI) operating at 70 eV. The column used for all analyses was a ZB-1701 GC capillary column (30 m × 250 μm id × 0.15 μm with 5 m guard column, Phenomenex). The analysis parameters were set according to Smart at al. [52]. Samples were injected under pulsed splitless mode with the injector temperature at 290°C. The helium gas flow through the GC-column was set at 1 mL^.^min^−1^. The interface temperature was set to 250°C and the quadrupole temperature was 200°C.

### Metabolomic data analysis

AMDIS software was used for deconvoluting GC-MS chromatograms and identifying metabolites using an in-house MCF mass spectra library. Identifications were based on both the MS spectrum of the derivatised metabolite and its respective chromatographic retention time. The relative abundance of identified metabolites was determined by ChemStation (Agilent) by using the GC base-peak value of a selected reference ion. These values were normalised by the biomass content in each sample as well as by the abundance of internal standard (2,3,3,3-d4-alanine). Samples were prepared in triplicates from two identical bioreactors for each strain and substrate.

## Abbreviations

PEG: Polyethylene glycol; PGM: Phosphoglucomutase; pgm: Phosphoglucomutase gene; TAL: Transaldolase; tal: Transaldolase gene; vvm: Gas volume flow per liquid volume per minute.

## Competing interests

The authors declare that they have no competing interests.

## Authors’ contributions

GEA designed and performed the molecular work and the strain selection, participated in the fermentation work and the metabolome analysis and drafted the manuscript. EK participated in the fermentation work and metabolome analysis. DM contributed in drafting the manuscript and the metabolome analysis. SVB performed the metabolome analysis. DGH contributed in the designing of the project. PC conceived the idea, designed the project and contributed in revising the manuscript. All authors read and approved the final manuscript.

## Supplementary Material

Additional file 1: Table S1 Relative *pgm* and *tal* transcription levels of the transformants, in comparison to the wild type strain (F3). Values were corrected according to the relative levels of total RNA, as they are represented by the density and intensity of the agarose gel bands, processed with ImageJ (NIH, Bethesda, ML, USA). **Table S2.** Arithmetic values of the specific activity of phosphoglucomutase and transaldolase for the transformed strains, compared to the wild type (F3), when grown in liquid cultures with glucose as carbon source. Standard deviation was lower than 3%. **Table S3.** Relative intracellular concentrations of amino and non-amino organic acids during aerobic growth of *F. oxysporum* F3 and FF11 strains on glucose and xylose. All values are average of three sample preparations from two bioreactors for each strain and carbon source. Standard deviation was less than 5%. Click here for file
