# Supplementary material for: Constitutive homologous expression of phosphoglucomutase and transaldolase increases the metabolic flux of Fusarium oxysporum
Source: Microb Cell Fact. 2014 Mar 20;13:43. doi: 10.1186/1475-2859-13-43 (PMC3999909; doi:10.1186/1475-2859-13-43)
Supplement: Additional file 1: Table S1 — Relative pgm and tal transcription levels of the transformants, in comparison to the wild type strain (F3). Values were corrected according to the relative levels of total RNA, as they are represented by the density and intensity of the agarose gel bands, processed with ImageJ (NIH, Bethesda, ML, USA). Table S2. Arithmetic values of the specific activity of phosphoglucomutase and transaldolase for the transformed strains, compared to the wild type (F3), when grown in liquid cultures with glucose as carbon source. Standard deviation was lower than 3%. Table S3. Relative intracellular concentrations of amino and non-amino organic acids during aerobic growth of F. oxysporum F3 and FF11 strains on glucose and xylose. All values are average of three sample preparations from two bioreactors for each strain and carbon source. Standard deviation was less than 5%. [file 1475-2859-13-43-S1.pdf]

Additional file 1

Table S1: Relative *pgm* and *tal* transcription levels of the transformants, in comparison to the wild type strain (F3). Values were corrected according to the relative levels of total RNA, as they are represented by the density and intensity of the agarose gel bands, processed with ImageJ (NIH, Bethesda, ML, USA).

	pgm	*tal*
F3 (wt)	1.00	1.00
FF1	2.46	5.02
FF2	4.01	1.32
FF3	8.92	1.36
FF4	7.43	2.19
FF5	2.65	5.89
FF6	11.64	3.59
FF7	11.95	1.48
FF8	5.46	1.63
FF10	4.77	5.73
FF11	10.16	6.03
FF12	11.65	1.25

Table S2: Arithmetic values of the specific activity of phosphoglucomutase and transaldolase for the transformed strains, compared to the wild type (F3), when grown in liquid cultures with glucose as carbon source. Standard deviation was lower than 3%.

	PGM		TAL	
	U/g DCW	U/g protein	U/g DCW	U/g protein
F3 (wt)	19.2	889.7	4.4	191.1
FF1	44.5	1781.2	17.5	1077.8
FF2	38.7	1215.1	3.6	205.9
FF3	11.6	1178.4	0.5	23.2
FF4	27.5	1284.7	1.9	45.7
FF5	15.3	721.8	43.8	1799.2
FF6	102.3	4287.1	36.6	1704.6
FF7	69.8	3125.5	15.7	623.9
FF8	70.7	3185.2	10.8	571.8
FF10	26.4	988.3	50.7	2198.9
FF11	71.6	3910.0	49.1	1890.8
FF12	57.2	2573.4	3.8	164.5

Table S3: Relative intracellular concentrations of amino and non-amino organic acids during aerobic growth of *F. oxysporum* F3 and FF11 strains on glucose and xylose. All values are average of three sample preparations from two bioreactors for each strain and carbon source. Standard deviation was less than 5 %.

	**Glucose**		**Xylose**	
	**F3**	**FF11**	**F3**	**FF11**
**Pyruvate metabolism**				
Pyruvate	n.d.*	n.d.	n.d.	n.d.
Lactate	68.6	205.7	100.9	129.1
Valine	147.1	5.8	1.2	1.1
Leucine	73.9	176.8	53.7	74.4
Alanine	221.0	347.7	302.2	196.9
**Glycine, serine, threonine metabolism**				
Glycine	118.4	273.7	104.7	137.4
Serine	4.9	11.7	4.2	3.9
Cysteine	0.7	1.1	0.6	0.4
Threonine	44.9	100.5	47.2	32.5
Aspartate	90.4	146.7	89.2	43.1
Asparagine	22.2	96.2	57.2	49.4
**Phenylalanine, tyrosine and tryptophan metabolism**				
Tryptophan	5.3	18.1	5.2	11.1
Tyrosine	4.6	29.7	13.3	17.6
Phenylalanine	4.2	13.3	7.8	10.5
Phosphoenolopyruvate	n.d.	n.d.	0.3	0.1
**Histdine metabolism**				
Histidine	17.3	37.4	16.6	17.7
**TCA cycle**				
Fumarate	27.6	34.4	24.6	13.4
Succinate	64.9	134.9	40.2	45.1
Malate	43.2	62.2	26.8	28.5
Citrate	215.3	310.5	267.7	171.9
γ-amino-*n*-butyric acid	41.6	91.3	47.9	66.2
Cis-aconitate	27.5	54.3	16.5	10.8
**Glutamine and glutamate metabolism**				
Lysine	40.1	64.9	35.5	44.3
L-2-Amino-adipate	0.9	1.6	1.2	1.3
Pyroglutamate	104.4	189.1	140.4	94.8
Glutamate	96.1	188.1	1.5	103.4
Glutamine	8.8	24.0	9.5	9.4
N-acetyl-l-glutamate	17.6	28.4	4.4	10.9
Proline	202.7	286.7	302.2	180.4
Ornithine	84.3	129.4	103.3	79
Glutathione	13.8	33.6	29.9	21.9
**Dibasic acid metabolism**				
Citramalate	1.2	1.5	0.9	1.5

Itaconate	13.6	31.9	6.4	5.4
Nicotinate	4.3	5.3	5.5	3.6
**Other metabolites**				
Myristate	1.1	2.1	2.4	1.1
L-Cystathionine	4.8	15.2	4.6	5.6

*n.d.: not detected
